# Developmental origins of brain disorders: roles for dopamine

**DOI:** 10.3389/fncel.2013.00260

**Published:** 2013-12-19

**Authors:** Kelli M. Money, Gregg D. Stanwood

**Affiliations:** ^1^Neuroscience Graduate Program, Vanderbilt UniversityNashville, TN, USA; ^2^Vanderbilt Medical Scientist Training Program, Vanderbilt UniversityNashville, TN, USA; ^3^Department of Pharmacology, Vanderbilt UniversityNashville, TN, USA; ^4^Vanderbilt Kennedy Center for Research on Human Development, Vanderbilt UniversityNashville, TN, USA

**Keywords:** neurodevelopment, frontal cortex, striatum, D1 receptor, D2 receptor, neuropsychiatric disease, dendrite, migration

## Abstract

Neurotransmitters and neuromodulators, such as dopamine, participate in a wide range of behavioral and cognitive functions in the adult brain, including movement, cognition, and reward. Dopamine-mediated signaling plays a fundamental neurodevelopmental role in forebrain differentiation and circuit formation. These developmental effects, such as modulation of neuronal migration and dendritic growth, occur before synaptogenesis and demonstrate novel roles for dopaminergic signaling beyond neuromodulation at the synapse. Pharmacologic and genetic disruptions demonstrate that these effects are brain region- and receptor subtype-specific. For example, the striatum and frontal cortex exhibit abnormal neuronal structure and function following prenatal disruption of dopamine receptor signaling. Alterations in these processes are implicated in the pathophysiology of neuropsychiatric disorders, and emerging studies of neurodevelopmental disruptions may shed light on the pathophysiology of abnormal neuronal circuitry in neuropsychiatric disorders.

## INTRODUCTION

Brain development requires a complex interplay of genetic and environmental factors. Disruption of these elements can affect neuronal structure, function, or connectivity and can alter developmental trajectory. In turn, this can lead to long-lasting, even permanent, effects and contribute to brain disorders later in life (Finlay, 2001; Lewis and Levitt, 2002; Frederick and Stanwood, 2009; Thompson et al., 2009; Rapoport et al., 2012; Ben-Ari, 2013). Neurodevelopmental alterations in the frontal/prefrontal cortex and striatum, which are both heavily involved in cognition, memory, emotion, and learning, are likely involved in the etiology of neuropsychiatric disorders like autism, substance use disorders, and schizophrenia (Simpson et al., 2010; Langen et al., 2012; Jonas et al., 2013). These brain regions receive prominent dopamine (DA) innervations and express DA receptors (Bjorklund and Dunnett, 2007; Tritsch and Sabatini, 2012; Roeper, 2013). In fact, DA and other neurotransmitters are expressed even prior to synaptogenesis, and activation of their receptors during development alters brain structure and connectivity with enduring anatomical and behavioral effects through adulthood (Schmidt et al., 1996; Spencer et al., 1998; Stanwood and Levitt, 2001, 2007; Stanwood et al., 2005; Bhide, 2009; Lu et al., 2009; Zhang et al., 2010; Bellone et al., 2011; Hamilton et al., 2011; McCarthy et al., 2011). However, the cellular and functional consequences of these activities are still poorly understood, and their linkages to specific diseases are often indirect and poorly elucidated.

Here we will review evidence for the role of DA receptors in neurodevelopmental processes with an emphasis on how DA D1 and D2 receptors modulate neuronal morphology in the frontal cortex and striatum. We will then briefly discuss the relevant intracellular signaling mechanisms through which DA receptors modulate neuronal morphology, and conclude with the implications of altered DA signaling on our understanding of the etiology and pathophysiology of neuropsychiatric disorders.

## ONTOGENY OF DOPAMINERGIC INNERVATION AND RECEPTOR EXPRESSION PATTERNS

DA innervation and receptor expression are present early in development, mature during adolescence, and form stable patterns in young adulthood. This prolonged developmental timeline provides a large window of “critical periods” during which potential disruptors can induce varied effects. For example, a landmark study by Andersen et al. (2002) showed that repeated exposure to methylphenidate (Ritalin) during the juvenile period reduces cocaine reward in adulthood, whereas adult methylphenidate exposure does not confer this protection.

Alterations in DA innervation patterns and receptor expression have been found in several brain disorders, including psychiatric disease. A decrease in DAergic axons in deep layers of medial prefrontal cortex and decreased D1 receptor binding occurs in schizophrenic patients (Okubo et al., 1997; Akil et al., 1999). In addition, increased D2 receptor binding in the striatum has been observed in patients with ADHD and depression (D’Haenen and Bossuyt, 1994; Shah et al., 1997). An understanding of the developmental timeline of the DA system and points at which the normal developmental trajectory can be altered are crucial to explore the implications of DAergic disruption.

The frontal cortex and striatum are innervated by DA-containing axons from the mesencephalic DA nuclei by mid-to-late gestation in rodents (Verney et al., 1982; Kalsbeek et al., 1988; Voorn et al., 1988), the predominant animal models used to study the developmental roles of DA. Voorn et al. (1988) demonstrated that DA innervation develops in well-defined spatiotemporal gradients in the lateral ganglionic eminence (presumptive striatum) that begins by embryonic day (E) 14 and coincides with striatal neurogenesis and cell differentiation. The rat developing cortex receives DAergic fibers that pass through the striatum, with innervation occurring in a lateral (E16) to medial fashion (E19). At birth, DAergic fibers are concentrated in the developing layer VI of frontal cortex. Fiber density increases over time in the deep layers of cortex with some fibers innervating more superficial layers in discrete cortical regions as the more superficial cortical layers develop (Kalsbeek et al., 1988). Mature DAergic innervation patterns are established in both regions by postnatal day 60. Studies in other species suggest that these sequences of events are very well conserved evolutionarily, although the exact timings are of course quite different based on gestation length (see below).

Dopamine receptor expression develops in concert with DAergic innervation. DA receptors are part of the G-protein coupled receptor superfamily and can be classified into two subgroups based on sequence, pharmacology, and G protein signaling: D1-like, which includes D1 and D5 receptors, and D2-like, which includes D2, D3, and D4 receptors (Missale et al., 1998; Beaulieu and Gainetdinov, 2011). Even before DAergic afferents have fully occupied the striatum or cortex, the main two DA receptor subtypes (D1 and D2 receptors) are already present (Schambra et al., 1994; Araki et al., 2007; Sillivan and Konradi, 2011). Higher levels of expression for both subtypes are found in the striatum compared to frontal cortex from development to adulthood (Araki et al., 2007; Sillivan and Konradi, 2011), and the D1 receptor subtype is expressed at higher levels than the D2 receptor subtype in both regions (Boyson et al., 1986; Neisewander et al., 1991; Couppis et al., 2008). D3, D4, and D5 receptors are consistently expressed at lower levels but still are present even during prenatal development. For example, D3 and D5 receptor mRNA is first found in the striatum at E12 and frontal cortex by E15 (Araki et al., 2007). However, D4 receptor mRNA, which is also present in the striatum at E12 and frontal cortex by E15, has its highest expression during embryonic development (Araki et al., 2007). D4 receptor mRNA decreases after birth with a more significant drop in expression seen in the frontal cortex (Nair and Mishra, 1995). D3 receptors are also are expressed transiently within the early postnatal somatosensory cortex during a key critical period of plasticity (Gurevich and Joyce, 2000; Gurevich et al., 2001), although their function there remains unknown.

**FIGURE 1:**
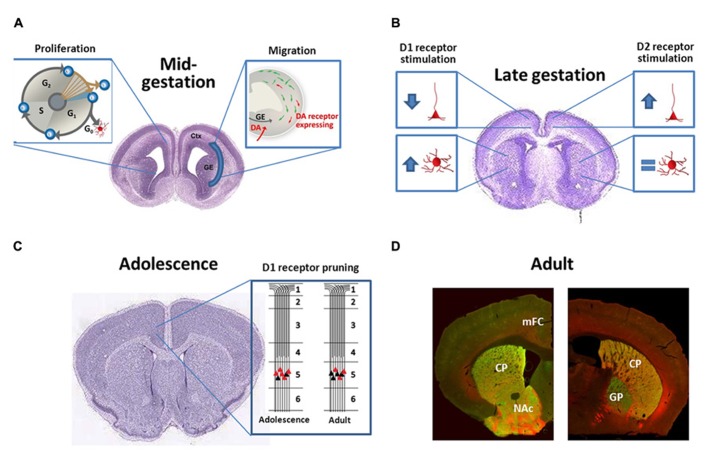
**(A)** During midgestation DA receptors have subtype-specific effects both on neuronal proliferation and interneuron migration; **(B)** During late-gestation DA receptors continue to be bioactive and regulate dendritic complexity of both cortical pyramidal neurons and striatal medium spiny projection neurons in a subtype-specific manner; **(C)** DA receptor expression typically peaks during adolescence and then declines. For D1 receptors in the frontal cortex, this is not just a reduction in total receptors per neuron, but rather a “pruning” of a subpopulation of D1 receptors that are transiently expressed on the terminals of cortico-accumbens neurons (Andersen et al., 2000; Brenhouse et al., 2008); **(D)** Photomicrographs demonstrate the localization of D1 [red, *Drd1*-tdTomato reporter (Ade et al., 2011)] and D2 [*Drd2*-eGFP reporter (Gong et al., 2003)] receptors in the rostral (left) and caudal (right) striatum of the adult mouse. Note the heavy labeling of D1 and D2 expressing cells within the CPu and NAc (note very few of these neurons co-express both receptors). More caudally (right) eGFP-labeled terminals can be visualized within the GP, representing the D2+ indirect pathway. D1+ axons, in contrast, bundle ventromedially to the GP and will eventually terminate in the substantia nigra and ventral tegmental area. CPu, caudate-putamen; Ctx, cortex; GE, ganglionic eminences; GP, globus pallidus, mFC, medial frontal cortex, and NAc, nucleus accumbens. The brain images in panels **(B)** and **(C)** are courtesy of the Allen Developing Mouse Brain Atlas and are available from: .

Dopaminergic development continues postnatally and impacts forebrain synaptogenesis and connectivity. For example, D1 receptor pruning is specifically associated with maturation of the excitation-inhibition balance in frontal cortex during adolescence (Andersen et al., 2000). Jung and Bennett (1996) demonstrated that D1 receptor expression steadily declines after the fourth postnatal week, which has been confirmed by others and is illustrated in **Figure 1C** (Andersen et al., 2000; Araki et al., 2007; Brenhouse et al., 2008). Cortical circuits and processing relies on connectivity and excitability of both excitatory glutamatergic pyramidal neurons and inhibitory GABAergic interneurons. Disruptions of excitation-inhibition balance in the frontal cortex have been reported in patients with schizophrenia, autism, major depressive disorder, and obsessive compulsive disorder (Harada et al., 2011; Cornew et al., 2012; Radhu et al., 2013), and many of these patients present with a first episode around adolescence. Risk of substance abuse disorders also increases during adolescence (O’Brien and Anthony, 2005). Genetically mediated DA variability has been suggested to shape complex behaviors emerging in adolescence, such as impulsivity and sensation seeking (Padmanabhan and Luna, 2013). DAergic regulation of frontal cortex output to subcortical areas mediates drug seeking behavior, with D1 receptor activation enhancing cortically driven drug seeking (Piazza et al., 1991; Kalivas et al., 2005). Moreover, cortical output neurons to the nucleus accumbens, a subcortical reward area, express higher levels of D1 receptors during adolescence in rodent models. This correlates with increased sensitivity to cocaine conditioned place preference. Brenhouse et al. (2008) found this increased preference to be enhanced with microinjections of D1-like receptor agonist in the prefrontal cortex but blunted with D1-like receptor antagonist, demonstrating the importance of D1 receptor signaling in heightened adolescent sensitivity to cocaine preference. This work demonstrates how the prolonged development of the DA system allows for vulnerability into adolescence. Thus, inappropriate alterations in D1 receptor signaling during adolescent cortical maturation could play a significant role in the development of neuropsychiatric diseases.

Phylogenetic differences in brain development exist between rodents and higher mammals (Workman et al., 2013). In regards to DA, monkeys and humans have more widespread cortical DA input than rodents and significantly denser DAergic innervation in motor, premotor, and supplementary motor association cortices (Berger et al., 1988, 1991; Gaspar et al., 1989; Meador-Woodruff et al., 1996). Human DAergic innervation occurs early in development and is strongly established by mid-gestation (Olson et al., 1973; Verney et al., 1991, 1993; Zecevic and Verney, 1995). The density of DAergic fibers to the prefrontal cortex in non-human primates increases during preadolescence, peaks during adolescence, and then declines (Rosenberg and Lewis, 1995). This evolution of the cortical DAergic system in primates suggests a more substantial role for DAergic innervation in development in more evolved species. The increased influence of DA in the human brain is exemplified by the increase in neuropsychiatric disease onset during adolescence, the time period when the DA system matures. The role of DA system development in neuropsychiatric disease vulnerability is an active area of research, and hopefully, the field will soon understand how to correct aberrant DA development in these vulnerable individuals.

## STRIATAL AND CORTICAL DOPAMINERGIC PATHWAYS

**FIGURE 2:**
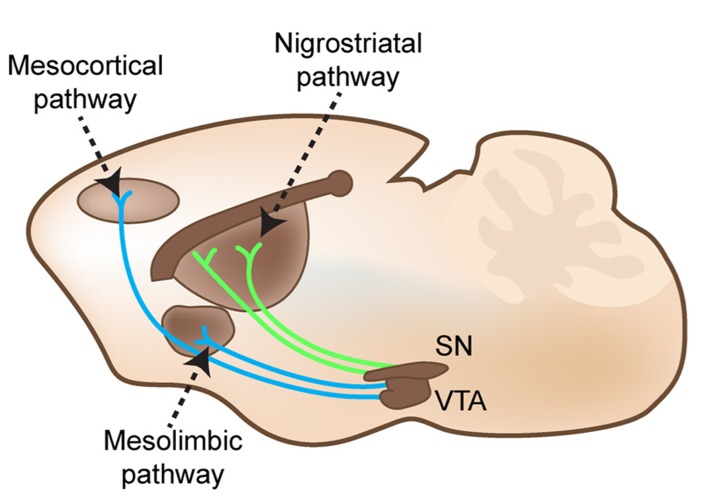
**Nigrostriatal, mesocortical, and mesolimbic pathways cartooned in an adult mouse brain in the sagittal plane.** Substantia nigra pars compacta (SN) projects to GABAergic projection neurons in the dorsal striatum. The ventral tegmental area (VTA) projects to both subcortical limbic areas and to the medial frontal cortex.

The striatum receives DAergic innervation from the substantia nigra pars compacta, which is known as the nigrostriatal pathway (shown in **Figure 2**). These DAergic afferents synapse on two distinct populations of GABAergic projection neurons, which express either D1 or D2 receptors (demonstrated in **Figure 1D**; Shuen et al., 2008; Santana et al., 2009; Ade et al., 2011). These populations send projections directly to or indirectly through the globus pallidus (external globus pallidus in primates) to the substantia nigra pars reticulata and endopeduncular nucleus (internal globus pallidus in primates) to make up the direct (D1 receptor expressing neurons) and indirect (D2 receptor expressing neurons) pathways to modulate movement and goal-oriented behavior (Missale et al., 1998; Beaulieu and Gainetdinov, 2011). Dysfunction within these pathways is a hallmark feature of multiple movement disorders, such as Parkinsonism and Huntingdon’s disease. Medications that target DA receptors can also modulate these pathways. For example, typical antipsychotics modulate this system by blocking D2 receptors; this action treats positive symptoms but can also lead to tardive dyskinesia, a serious side effect characterized by involuntary, repetitive movements. The importance of the nigrostriatal DAergic projections onto the direct and indirect pathway projection neurons is made evident by the devastating symptoms that result from disruption of this pathway, but other DAergic pathways can also play an important role in disease.

The ventral tegmental area (VTA), another mesencephalic nucleus, gives rise to two additional DAergic pathways. Projections from the VTA to limbic areas, such as the nucleus accumbens, and to the frontal cortex form the mesolimbic and mesocortical pathways (shown in **Figure 2**), respectively. In the nucleus accumbens, patterns of D1 and D2 receptor expression are similar to the dorsal striatum (demonstrated in **Figure 1D**). In addition, the shell of the nucleus accumbens expresses high levels of D3 receptors (Le Moine and Bloch, 1995). DA receptor expression is lower in the frontal cortex and found in several types of neurons. In the frontal cortex, D1 receptors appear to be the most prominent DA receptor, and both D1 and D2 receptors are found in deep layer V/VI glutamatergic projection neurons and parvalbumin-containing GABAergic interneurons (Le Moine and Gaspar, 1998; Muly et al., 1998). D4 and D5 receptors are expressed at low levels in frontal cortex (Beaulieu and Gainetdinov, 2011). Importantly, these pathways and DA receptor expression patterns have great functional significance. The mesolimbic pathway is mainly associated with reward, while the mesocortical pathway is associated with cognitive function. Aberrant reward processing drives substance abuse disorders, and cognitive dysfunction exists as an often overlooked but devastating feature of many neuropsychiatric disorders (Carter et al., 1998; Bosboom et al., 2004; Medalia and Lim, 2004). Studies of imbalanced mesolimbic and mesocortical DAergic signaling support a potential therapeutic role for DA in reward and cognitive dysfunction in neuropsychiatric disease (Murphy et al., 1996; Volkow et al., 1998; Goldman-Rakic et al., 2004). Thus, DA can influence multiple aspects of human behavior both directly through striatal projections and indirectly through alterations in reward and cognition (for description of the trajectories of growing DA axons and signals contributing to topography, see Prestoz et al., 2012).

## DEVELOPMENTAL EFFECTS OF DOPAMINERGIC MODULATION

DA is one of the earliest neurotransmitters expressed in the developing brain and plays a significant role in the development of neuronal cytoarchitecture. The presence of DAergic projections to the developing cortex and striatum, as well as DA receptors on precursor cells, places DA in an ideal position to modulate neuronal development, including cell proliferation, migration, and differentiation all of which can lead to altered connectivity and dysfunctional synapses. In fact, genetic and pharmacologic models of disrupted DA signaling display changes in cortical and striatal circuitry (Stanwood et al., 2001b, 2005; Song et al., 2002; Kellendonk et al., 2006; McCarthy et al., 2007; Zhang et al., 2010). Environmental, social, and inflammatory challenges during sensitive periods of forebrain development also produce neuro(mal)adaptions in these regions and phenocopy some aspects of brain disorders (Di Forti et al., 2007; Kern et al., 2010; Han et al., 2012; Selemon and Friedman, 2013). For example, models of prenatal infection, stress, and exposure to drugs of abuse show long-lasting changes in DA receptor expression and function and have been associated with increased risk for psychiatric disorders (Alonso et al., 1994; Stanwood et al., 2001b; Berger et al., 2002; Zuckerman et al., 2003; Andersen and Teicher, 2009; Bhide, 2009; Brown et al., 2012). Taken together, these data have led our group and others to hypothesize that DAergic modulation of developmental processes produces long lasting alterations that contribute to cortical and striatal dysfunction in neuropsychiatric disorders.

### NEUROPROGENITOR PROLIFERATION

The earliest observed developmental effects of altered DA signaling are changes in neuroprogenitor proliferation. Selective D1-like receptor agonists can decrease incorporation of the cell cycle marker bromodeoxyuridine (BrdU) in the developing medial frontal cortex and lateral ganglionic eminence, a basal forebrain progenitor pool that is the source for most striatal neurons (illustrated in **Figure 1A**). Decreased BrdU incorporation is indicative of less neuronal progenitors undergoing division. Both D1-like and D2-like receptors contribute to neurogenesis but with opposite effects; blockade of D1-like receptors and activation of D2-like receptors increase the number of neuronal progenitors undergoing cell division (Ohtani et al., 2003; Popolo et al., 2004). Administration of the DA precursor L-DOPA during early gestation decreases cell proliferation (McCarthy et al., 2007), suggesting that D1 receptor mediated effects may dominate *in vivo*. Although these studies did not mechanistically investigate how D1 and D2 receptors alter BrdU incorporation, another group examined receptor-induced changes in cell cycle protein expression. Indeed, D1-like receptor activation promotes downregulation of cyclin D and upregulation of the cyclin dependent kinase inhibitor p27Kip1 (Zhang et al., 2005). Cyclin D promotes the transition from the growth phase to S-phase of the cell cycle while p27Kip1 inhibits it (Sheaff and Roberts, 1998). In addition, Raf-1, a component of the mitogen-activated protein kinase (MAPK) pathway that promotes mitosis, is downregulated by D1-like receptor activation (Denhardt, 1999; Zhang et al., 2005). The mechanism by which these effects on cell cycle proteins occur is not known, although it appears to be PKA-independent (Zhang et al., 2005). The effects of transient differences in cell cycle proteins have yet to be understood, but small deviations in the number of cells available to release or receive important developmental signals could significantly alter developmental trajectory. And although it is beyond the scope of our current review, it is also worth noting that both acute and chronic DA depletion decreases subependymal neuronal proliferation (Lewis et al., 1977; Patel et al., 1977), and DA receptors, especially D3 receptors, can guide adult neurogenesis as well (Kim et al., 2010; Egeland et al., 2012; Lao et al., 2013).

### MIGRATION

As mentioned above, inappropriate excitation-inhibition balance in frontal cortex is found in several neuropsychiatric diseases (Harada et al., 2011; Cornew et al., 2012; Radhu et al., 2013), which could be partly due to a lack of adequate interneuron migration. The two main cell types in the frontal cortex, glutamatergic pyramidal projection neurons and GABAergic interneurons, are derived from two separate progenitor pools in the developing rodent forebrain. Pyramidal neurons are produced locally in the neuroepithelium of the developing cortex and migrate radially to the appropriate cortical layer. Concurrently, GABAergic interneurons generated in the basal forebrain in the medial and caudal ganglionic eminences migrate tangentially to the cortex (de Carlos et al., 1996; Wonders and Anderson, 2006). The number of GABAergic interneurons increases through mammalian brain evolution beyond what the ganglionic eminences can provide. Therefore, in primates, GABAergic interneurons are also produced in the dorsal forebrain and migrate radially through cortex (Letinic et al., 2002; Petanjek et al., 2009). However, this tangentially migrating population of interneurons is still a substantial portion of interneurons produced, and dysregulation of either population would contribute to neuropsychiatric disease (Hansen et al., 2013; Ma et al., 2013).

GABAergic neurons in the medial and caudal ganglionic eminences express DA receptors, and these forebrain regions receive DAergic innervation during the embryonic period when migration occurs. The Bhide lab has demonstrated that altering DA receptor signaling with prenatal cocaine or receptor-specific agonists/antagonists disrupts tangential migration (illustrated in **Figure 1A**). Prenatal cocaine exposure decreases tangential migration of cortical GABAergic neurons, perhaps mediated by the loss of D1 receptor signaling (Jones et al., 2000; Crandall et al., 2004; Stanwood and Levitt, 2007; McCarthy et al., 2011). In addition, application of D1-like receptor agonists in mouse embryonic slice cultures promotes cortical GABAergic neuron migration whereas D2-like receptor agonism decreases migration (Crandall et al., 2007). Overexpression of the D1 receptor by *in vitro* electroporation increases the effect of D1-like receptor agonists on promoting tangential migration (Crandall et al., 2007). The basis for opposing D1 vs. D2 receptor effects is not fully understood, but cytoskeletal reorganization must occur for neuronal migration. D1-like receptor stimulation leads to increased neurite localization of cytoskeletal elements needed for cell motility, such as cytoplasmic dynein motor protein and tubulin, whereas D2-like receptor stimulation leads to increased cell body localization of these cytoskeletal elements. Crandall et al. (2007) suggest that this subtype-specific redistribution of cytoskeletal elements needed for cell motility leads to opposite effects on neuronal migration. Elucidation of the molecular pathways responsible for receptor-dependent modulation of neuronal migration may provide new targets for the restoration of neurodevelopmental trajectory.

### DENDRITIC AND AXONAL GROWTH

Dendritic and axonal growth are activity-dependent, highly dynamic processes that remain partially plastic until adulthood (McAllister, 2000). Disruption of dendritic or axonal growth alters both the quantity and nature of neural connections. Not surprisingly, anomalies in brain connectivity have been linked to several neuropsychiatric diseases, including intellectual disability and schizophrenia (Kaufmann and Moser, 2000; Black et al., 2004). In fact, DA receptor activation modulates dendritic structure, creating subtle but significant changes in brain architecture that may increase vulnerability to neurological and/or neuropsychiatric disease.

In this regard, activation of DA receptors influences axonal and dendritic growth in a subtype-specific manner. Primary cultures have been the primary model to test the *in vitro* effects of DAergic modulation on neurite outgrowth, a term to describe both the developing axon and dendrite. Several groups demonstrated that D1-like receptor agonists significantly reduce neurite outgrowth in frontal cortical neuronal cultures (Reinoso et al., 1996; Song et al., 2002; Li et al., 2013), which is depicted in **Figure 1B**. Song et al. (2002) further observed that decreased neurite outgrowth is associated with destabilization of dendritic microtubule associated protein (MAP2) by increased phosphorylation. Increased MAP2 phosphorylation is commonly associated with decreased neurite outgrowth (Sanchez et al., 2000). However, activation of numerous kinases causes MAP2 phosphorylation, providing an effector but not the mechanism (Sanchez et al., 2000). An animal model of prenatal cocaine exposure also demonstrates the importance of cortical D1 receptor signaling to suppress neurite outgrowth. This model produces a long-lasting uncoupling of D1 receptor signaling and concomitant increase in neurite outgrowth (Jones et al., 2000; Stanwood and Levitt, 2007). Our group has also observed increased basal neurite outgrowth in cortical cultures derived from D1 receptor null embryos as compared to cultures derived from wild-type littermates (unpublished data). With regards to axonal effects, evidence suggests that D1-like receptor stimulation also alters cortical axon growth patterns by decreasing expression of the netrin-1 receptor (Sillivan, 2011). In contrast to the growth-suppressing effects of D1-like receptor stimulation, D2-like receptor activation in primary cortical cultures leads to an increase in neurite outgrowth (illustrated in **Figure 1B**; Todd, 1992; Reinoso et al., 1996). Thus, D1-like receptor stimulation decreases neurite outgrowth and netrin-1 receptor expression whereas D2-like receptor stimulation increases neurite outgrowth *in vitro*. Yet, the DA receptor-expressing population is only approximately 10–15% of neurons in the frontal cortex (Santana et al., 2009). Ongoing research in our group is testing whether these effects are cell-specific (only occurring in neurons expressing DA receptors) or if a non-cell specific mechanism leads to DA receptor-induced changes on neurite outgrowth in other subpopulations.

In contrast, application of D1-like receptor agonists to striatal neuronal cultures promotes neurite outgrowth, which can be blocked with D1-like receptor antagonists and is demonstrated in **Figure 1B** (Schmidt et al., 1996, 1998). This increased neurite outgrowth is associated with an increased number of growth cones and arborization (Schmidt et al., 1998). However, D2-like receptor activation shows no effect on striatal neurite outgrowth (also demonstrated in **Figure 1B**; Schmidt et al., 1996). Hence, D1-like receptor stimulation increases neurite outgrowth in the striatum *in vitro*.

Studies from intact brains bolster the importance of developmental DA receptor signaling on dendritic growth in DA-rich brain regions. Models of DA depletion (i.e., 6-OHDA treatment or mesencephalic lesion) show decreased expression of axon guidance- and cytoskeletal growth-related proteins as well as decreased length of pyramidal layer V basal dendrites in frontal cortex (Kalsbeek et al., 1989; Krasnova et al., 2007). In human studies, adolescents exposed prenatally to cocaine display changes in cortical thickness (Roussotte et al., 2012; Liu et al., 2013). MAP2 labeling of D1 receptor knockout mice and diolistic labeling of rabbits prenatally exposed to cocaine show abnormal apical dendrites of cortical pyramidal cells with decreased bundling and increased tortuous patterning (Jones et al., 2000; Stanwood et al., 2001a, 2005), which supports the increased basal neurite outgrowth observed *in vitro.* Conversely, silver impregnation of cortical neurons from D1 receptor overexpressing mice show decreased apical dendrite length compared to wild type littermates, which further supports a role for D1 receptor signaling to promote dendritic growth (Song et al., 2002). Finally, the Kellendonk lab has shown that mice overexpressing D2 receptors in the striatum have decreased complexity and length of striatal dendritic arbors (Cazorla et al., 2012). Although differences in normal striatal dendritic morphology have been studied based on receptor subtype expression (Gertler et al., 2008), to our knowledge the effects of reduced or absent D1 receptor signaling on striatal dendritic morphology has not been examined.

### SPINOGENESIS AND SYNAPTOGENESIS

Dendritic spinous processes start to form at the beginning of synaptogenesis during the first postnatal week and peak in adolescence but continue to be structurally modified throughout adulthood (Zhang and Benson, 2000). Spines develop from dendritic filopodia that interact with incoming axons, but some data suggest that dendritic spines can form independent of axonal interaction (Zhang and Benson, 2000; Whitford et al., 2002; Yuste and Bonhoeffer, 2004). Dendritic spines are critical for synaptic plasticity, allowing for compartmentalization of post-synaptic signaling and synaptic specialization (Yuste and Bonhoeffer, 2001). Additionally, alterations in spine density likely originating during development are found in many neuropsychiatric diseases, such as autism and schizophrenia (Kaufmann and Moser, 2000; Garey, 2010; Penzes et al., 2011; Glausier and Lewis, 2013; Seshadri et al., 2013). Importantly, DA plays a role in spinogenesis. For example, a functional hyperdopaminergia, which occurs in DA transporter (DAT) knockout mice, leads to a loss of proximal dendritic spines in striatal projection neurons (Berlanga et al., 2011). This model also shows a behavioral phenotype of hyperactivity and anxiety as well as altered mesocortical circuitry (Zhang et al., 2010; Carpenter et al., 2012), and DA dysregulation occurs not only during development, but continues into adulthood in this model. Primate and rodent models of DA depletion also show decreases in striatal dendritic spines (Ingham et al., 1989; Neely et al., 2007; Villalba et al., 2009). Thus, a delicate balance of DA signaling is required for formation/stabilization of dendritic spines. Cortical pyramidal neurons of D1 and D2 receptor knockout mice exhibit decreased dendritic spine density (Wang et al., 2009), and moderate D1-like and D2-like receptor activation both have been shown to increase spine density and spinophillin expression in the striatum *in vitro* (Fasano et al., 2013). Studies have not yet shown if higher concentrations of DAergic agonists, more like the activation levels seen in the hyperdopaminergic DAT knockout mouse, would phenocopy the decreased dendritic spine density seen in that (potentially non-physiological) mutant. In fact, during adolescence D2 receptor activation decreases striatal dendritic spines (Jia et al., 2013). Interestingly, the spine loss in cortical pyramidal neurons resulting from DA depletion can be ameliorated by spinogenesis-promoting antipsychotics like clozapine, providing further support for spine dysfunction in the pathology of neuropsychiatric disease (Critchlow et al., 2006; Wang and Deutch, 2008).

Since dendritic spines serve as the recipients of synaptic input and both develop concurrently, spinogenesis and synaptogenesis are intimately connected and are often co-regulated (Zhang and Benson, 2000; Whitford et al., 2002; Yuste and Bonhoeffer, 2004; Haas, 2006). Multiple points of vulnerability beyond spine formation can be altered to disrupt synaptogenesis. Axonal pathfinding must lead to the vicinity of the target neuron for initial contact to be made. Cell-cell adhesion then needs to occur, followed by pre- and post-synaptic differentiation and synapse strengthening (Garner et al., 2002). Synapse formation, when it occurs properly, leads to the formation of connections on a particular part (usually dendritic spines) of a target neuron, sometimes over very long distances (Südhof, 2006). Schizophrenia and other neurodevelopmental disorders are at least in part, diseases of altered synaptic connectivity. Inappropriate dendritic arborization, spinogenesis, and synaptic connections lead to inappropriate connectivity and altered excitation-inhibition balance (McGlashan and Hoffman, 2000; Spronsen and Hoogenraad, 2010; Duman and Aghajanian, 2012). This is supported by observations of decreased dendritic spines and synaptic labeling in post mortem cortex of schizophrenic patients (Glantz and Lewis, 1997, 2000; Honer et al., 1999). Thus, synaptogenesis can be altered by any of the previously discussed developmental events and plays an important role in neuropsychiatric disease pathology.

Synaptogenesis follows a similar timeline of development as spinogenesis and displays similar responses to DAergic modulation. Synapses begin to form during the first postnatal week (Hattori and McGeer, 1973). The number of synapses peak during adolescence, which is followed by synaptic pruning (Zhang and Benson, 2000). In the striatum and frontal cortex, electron microscopy has shown that DAergic axons form symmetric synapses similar to most GABAergic interneurons whereas glutamatergic pyramidal axons forms asymmetric synapses (Smiley et al., 1992; Antonopoulos et al., 2002; Yokofujita et al., 2008). Neonatal DA depletion decreases the number of symmetric synapses and changes the structure of asymmetric synapses (Onténiente et al., 1980; Tennyson et al., 1982; Ingham et al., 1991, 1993). Others have suggested that neonatal DA depletion can alter dendritic structures in the frontal cortex (Sherren and Pappas, 2005), and cause dysregulation of gene expression patterns required for normal brain architecture (Krasnova et al., 2007). DA receptor antagonists can decrease prefrontal synapse density whereas agonists slightly increased density (Sugahara and Shiraishi, 1998). Furthermore, haloperidol, a D2-like receptor antagonist, decreases hippocampal synaptic density *in vitro* (Critchlow et al., 2006). Opposing D1 vs. D2 receptor developmental effects are lost with regards to spinogenesis and synaptogenesis, and hopefully further study will produce a mechanistic explanation. DA lesion studies in adult animals support a continued role for DA in the maintenance of corticostriatal synapses in the striatum (for a comprehensive review of DA-regulated morphological and electrophysiological changes, please see Arbuthnott et al., 2000 and Surmeier et al., 2011). Taken together, these studies support a crucial role for DA in formation and stabilization of synaptic connections in the striatum and frontal cortex, providing further evidence for developmental disruptions of DA and other neurotransmitters in the structural pathology of neuropsychiatric disease.

## DOPAMINE D1 RECEPTOR SIGNALING MECHANISMS IN DEVELOPMENTAL PROCESSES

Establishing a detailed signaling mechanism for the above mentioned *in vitro* and *in vivo* effects on developmental processes is complicated by multiple signaling cascades and receptor interactions. While all five DA receptors likely contribute to these effects in specific ways, studies to date have predominantly examined D1 receptors, and thus our discussion will also focus there. In general, D1-like receptors transduce their signals by activating adenylyl cyclase and increasing intracellular cAMP via Gαs/Gαolf coupled proteins. Conversely, D2-like receptors typically transduce their signals by inhibiting adenylyl cyclase and decreasing cAMP (Missale et al., 1998; Beaulieu and Gainetdinov, 2011). Intracellular cAMP activates protein kinase A (PKA), which phosphorylates proteins such as DA and cAMP-regulated phosphoprotein (DARPP-32) and cAMP responsive-element binding protein (CREB; Parker et al., 1996; Greengard et al., 1999). CREB promotes transcription of genes with cAMP response elements. Additionally, MAPKs like extracellular signal-related kinase (ERK) are activated by D1 receptor stimulation and increase CREB signaling (Missale et al., 1998).

DA receptors also participate in multiple non-Gα protein signaling mechanisms. DA receptor mediated effects can involve Gβ/Gγ proteins as well as other receptors (Missale et al., 1998; Beaulieu and Gainetdinov, 2011). For example, D1 receptors interact with NMDA receptors (Lee et al., 2002; Nai et al., 2010), adenosine receptors (Fuxe et al., 2007; Shen et al., 2013), and neurotrophin TrkB receptors (Iwakura et al., 2008), among others. D2 receptors complex with adenosine A_2A_ receptors (Canals et al., 2003) and sigma-1 receptors (Navarro et al., 2013) and recruit protein kinase B/glycogen synthase kinase 3 (AKT/GSK3) signaling pathways via a slower, cAMP-independent signaling mechanism (Beaulieu et al., 2005, 2007). G protein-coupled receptor kinases and arrestins, normally associated with receptor desensitization, form a scaffold for D2 receptor interactions with protein phosphatase 2A and AKT. The resulting dephosphorylation of AKT increases activity of GSK3, a target of lithium and many antipsychotic drugs (Beaulieu et al., 2004; Li et al., 2007; Lovestone et al., 2007).

Given the profound differences between how neurites of cortical and striatal neurons respond to DA receptor agonists, it is likely that intracellular signals are regulated in cell-specific ways. These are difficult experiments to carry out, however, and we currently have a fairly superficial understanding of these mechanisms. Both D1-like receptor agonists and PKA activators increase cortical MAP2 phosphorylation, decreasing the ability of MAP2 to stabilize the dendritic cytoskeleton and thus decreasing neurite outgrowth (Song et al., 2002). Neurite outgrowth facilitation by D1-like receptor stimulation in the striatum is also PKA-dependent and at least in part CREB-mediated (Schmidt et al., 1998). Additionally, the Chao lab has demonstrated that increased neurite outgrowth in striatal cultures is at least partially dependent on D1 receptor-mediated neurotrophin TrkB receptor activation. Their study showed that D1-like receptor agonist treatment in primary striatal cultures leads to activation and increased surface expression of the TrkB receptor, and is accompanied by phosphorylation of downstream Trk signaling proteins (Iwakura et al., 2008). Thus, taken together, current evidence suggests that the D1-like receptor-mediated changes in neurite outgrowth are PKA-dependent in both cortex and striatum with an additional neurotrophin TrkB receptor-dependent component only in the striatum. The dichotomy between D1 and D2 receptor signaling in the context of developmental dysfunction needs to be understood in order to target the neurodevelopmental origins of neuropsychiatric disease. Furthermore, future studies should identify potential roles for differences in G protein coupling (e.g., Gαs in cortex and Gαolf in striatum Herve et al., 1993; Herve, 2011), and accessory proteins with differential expression patterns (e.g., GPR88 in striatum Logue et al., 2009; Van Waes et al., 2011; Marley et al., 2013).

## GENETIC DISRUPTIONS OF DA SIGNALING AND NEUROPSYCHIATRIC DISEASE

The DA system plays a fundamental role in psychotic disorders, with D2 receptor antagonism being a major target for both typical and atypical antipsychotics. While D2 receptor antagonism may be useful in controlling many of the positive symptoms in schizophrenia, emerging data implicates D1-like receptors as a target for negative symptoms in schizophrenia (Goldman-Rakic et al., 2004; Arnsten, 2013). In addition, several genetic perturbations in DA-related genes have been found in patients with neuropsychiatric disease. Patients with 22q11 deletion syndrome, which disrupts DA-related genes (e.g., catechol-O-methyltransferase, *COMT*) among others have an increased risk for neuropsychiatric disorders, including schizophrenia-like psychosis, autism, and anxiety disorders (Karam et al., 2010; Jonas et al., 2013). Hypomethylation of the *COMT* gene, which leads to an increase in the enzyme that breaks down DA, has been associated with schizophrenia and bipolar disorder (Abdolmaleky et al., 2006; Nohesara et al., 2011). The translocation in the gene disrupted in schizophrenia-1 (*DISC-1*) was originally found in a large Scottish family with abnormally high rates of neuropsychiatric disease (Millar et al., 2001). Interestingly, knockdown of *DISC-1* in animal models causes altered DAergic maturation and behavioral changes associated with altered prefrontal cortex circuitry, suggesting a role for DA in *DISC-1* associated neuropsychiatric disease (Niwa et al., 2010). Association studies implicate the A1 allele of the Taq1 polymorphism of *DRD2* in the development of Tourette’s syndrome, ADHD, autism, PTSD, and alcoholism (Comings et al., 1991). The Holden lab has found DA-related genes (*DRD1*, *DRD2*, and *PPP1R1B*) associated with the severity of autism in families with males only affected (Hettinger et al., 2008, 2012). Others have found polymorphisms in *DRD3* and *DRD4* associated with autism severity (de Krom et al., 2009; Gadow et al., 2010; Staal et al., 2012) and DAT polymorphisms linked to both ADHD and bipolar disorder (Sharp et al., 2009). Additionally, polymorphisms in the gene encoding the downstream signaling protein Akt are associated with both bipolar disorder and schizophrenia (Thiselton et al., 2008; Karege et al., 2012). While the effect sizes in these studies are not large, and the link to DA is sometimes indirect, taken together these findings emphasize that disrupted DAergic homeostasis may be a central mechanism in the risk of developing a neuropsychiatric disorder.

## CONCLUSION

There is strong evidence that DA plays a critical role in the generation of typical brain structure and function. DA and its cognate receptors are present in the striatum and frontal cortex early in brain development and contribute to the development of pathways needed for movement, cognition, and reward. Altered DA signaling can affect the proliferation, migration, and differentiation of specific subpopulations of neurons, and thus impact frontal cortex and striatal neurocircuitry. D1 and D2 receptor subtypes often have opposing effects on these developmental processes, and the underlying mechanism for this dichotomy as well as the signaling pathways that mediate these subtype-specific effects remain mostly unknown.

Yet, DA is not the only neuromodulator that plays a role in developmental processes. Serotonin is present before synaptogenesis (Hansson et al., 1998) and alters neurite outgrowth and axon pathfinding *in vitro* in a receptor-dependent manner (Lotto et al., 1999; Persico et al., 2006; Bonnin et al., 2007; Tajiri et al., 2012; Trakhtenberg and Goldberg, 2012; Anelli et al., 2013; Speranza et al., 2013; for comprehensive review of roles of serotonin in development, see van Kesteren and Spencer, 2003; Pino et al., 2004; Bonnin and Levitt, 2011; Trowbridge et al., 2011). In addition, decreased cortical thickness in reelin mutants is postulated to be driven by cortical cholinergic deprivation (Liu et al., 2001; Sigala et al., 2007), and both nicotinic and muscarinic cholinergic receptor manipulation alters neurite outgrowth (Lipton et al., 1988; Pugh and Berg, 1994; Small et al., 1995; for comprehensive review, see Ruediger and Bolz, 2007; Dwyer et al., 2009; Bruel-Jungerman et al., 2011). Finally, noradrenergic neurons are thought form early synapses in the cortical plate and mediate neuronal migration and laminar formation (Herlenius and Lagercrantz, 2001). DA thus does not provide a singular role in these developmental processes, and future studies will need to address additional neuromodulators in combinatorial patterns.

Our understanding of the role of developmental disruptions of DA in the pathology of neuropsychiatric disorders is still maturing. DA-rich areas, such as the prefrontal cortex and striatum, are clearly dysfunctional in neuropsychiatric disorders like schizophrenia, substance use disorders, and ADHD (Arnsten, 2006, 2011; Leppanen, 2006; Andersen and Teicher, 2009; Arnsten et al., 2012). Developmental alterations in dendritic growth and/or GABAergic interneuron migration likely contribute to the development of pathology in these disorders (Beasley and Reynolds, 1997; Kaufmann and Moser, 2000; Black et al., 2004; Curley et al., 2011). The increased DAergic influence in the cortex of primates and dysfunction of DA-rich cortical areas in neuropsychiatric disorders suggests an evolutionally increased role of DA, which has also resulted in an evolutionarily increased vulnerability to disease. A new era of neuropsychiatric pharmacologic targets should address the underlying pathology resulting from developmental disruptions in DA and other neurotransmitters. Importantly, early interventions have the most positive impacts in neurodevelopmental disorders and learning disabilities (Bradshaw et al., 2012); our hope is that a detailed mechanistic understanding of brain substrates and developmental pharmacology will allow clinicians to make similar rational interventions within neuropsychiatry.

## Conflict of Interest Statement

The authors declare that the research was conducted in the absence of any commercial or financial relationships that could be construed as a potential conflict of interest.
